# Safety, pharmacokinetics, and antitumor properties of anlotinib, an oral multi-target tyrosine kinase inhibitor, in patients with advanced refractory solid tumors

**DOI:** 10.1186/s13045-016-0332-8

**Published:** 2016-10-04

**Authors:** Yongkun Sun, Wei Niu, Feng Du, Chunxia Du, Shuting Li, Jinwan Wang, Li Li, Fengqing Wang, Yu Hao, Chuan Li, Yihebali Chi

**Affiliations:** 1 Department of Medical Oncology, Cancer Hospital, Chinese Academy of Medical Sciences and PUMC, Beijing, 100021 China; 2 The State Key Laboratory of Drug Research, Shanghai Institute of Materia Medica, Chinese Academy of Sciences, Shanghai, 201203 China; 3 The VIPII Gastrointestinal Cancer Division of Medical Department, Peking University Cancer Hospital and Institute, Beijing, 100142 China; 4 School of Public Health, Nanjing Medical University, Nanjing, China

**Keywords:** Anlotinib, Anti-angiogenesis, Phase I study, Advanced refractory solid tumors, Pharmacokinetics, Safety

## Abstract

**Background:**

Anlotinib is a novel multi-target tyrosine kinase inhibitor that is designed to primarily inhibit VEGFR2/3, FGFR1-4, PDGFR α/β, c-Kit, and Ret. We aimed to evaluate the safety, pharmacokinetics, and antitumor activity of anlotinib in patients with advanced refractory solid tumors.

**Methods:**

Anlotinib (5–16 mg) was orally administered in patients with solid tumor once a day on two schedules: (1) four consecutive weeks (4/0) or (2) 2-week on/1-week off (2/1). Pharmacokinetic sampling was performed in all patients. Twenty-one patients were further enrolled in an expanded cohort study on the recommended dose and schedule. Preliminary tumor response was also assessed.

**Results:**

On the 4/0 schedule, dose-limiting toxicity (DLT) was grade 3 hypertension at 10 mg. On the 2/1 schedule, DLT was grade 3 hypertension and grade 3 fatigue at 16 mg. Pharmacokinetic assessment indicated that anlotinib had long elimination half-lives and significant accumulation during multiple oral doses. The 2/1 schedule was selected, with 12 mg once daily as the maximum tolerated dose for the expanding study. Twenty of the 21 patients (with colon adenocarcinoma, non-small cell lung cancer, renal clear cell cancer, medullary thyroid carcinoma, and soft tissue sarcoma) were assessable for antitumor activity of anlotinib: 3 patients had partial response, 14 patients had stable disease including 12 tumor burden shrinkage, and 3 had disease progression. The main serious adverse effects were hypertension, triglyceride elevation, hand-foot skin reaction, and lipase elevation.

**Conclusions:**

At the dose of 12 mg once daily at the 2/1 schedule, anlotinib displayed manageable toxicity, long circulation, and broad-spectrum antitumor potential, justifying the conduct of further studies.

**Electronic supplementary material:**

The online version of this article (doi:10.1186/s13045-016-0332-8) contains supplementary material, which is available to authorized users.

## Background

Several molecular pathways play crucial roles in cancer development, including proliferation pathways, cell-cycle control pathways, and the processes of angiogenesis [1, 2], where some important mediators have been successfully identified as potential targets for new anticancer treatments. Multi-targeted tyrosine kinase inhibitors (TKIs) have demonstrated significant antitumor effects in a variety of tumor types through the inhibition of angiogenetic and proliferative signaling [3–5].

Anlotinib is a novel oral multi-targeted receptor tyrosine kinase inhibitor, which has a broad spectrum of inhibitory action on tumor angiogenesis and growth. The vascular endothelial growth factor (VEGF) isoforms and their receptors (VEGFRs) are crucial proteins in both vasculogenesis and angiogenesis and have been proved as effective anticancer target [3, 5, 6]. In vitro studies using recombinant enzymes indicated that anlotinib selectively inhibited VEGFR2/KDR and VEGFR3 approximately 20 and 500 times as potent as sunitinib and sorafenib, respectively (details pending publication elsewhere). On the other hand, dysregulation of fibroblast growth factor (FGF)/fibroblast growth factor receptor (FGFR) axis results in aggressive cancer phenotypes by promoting cancer progression and enhancing the angiogenic potential of tumor microenvironment [7–9]. FGF/FGFR signaling alterations has been connected with chemotherapy resistance and poor clinical outcome [10–13]. Preclinical results showed that anlotinib significantly inactivated FGFR1-4, especially the FGFR2, to a greater extent than sorafenib did.

Anlotinib suppressed tumor cell proliferation via inhibition of platelet-derived growth factor receptors α/β (PDGFR α/β), c-Kit, Ret as well as Aurora-B, c-FMS, and discoidin domain receptor 1(DDR1), which was a group of newly identified kinase targets involving the tumor progression [14–17]. In addition, anlotinib showed antitumor activity against tumor cells carrying mutations in PDGFR α, c-Kit, Met, and epidermal growth factor receptor (EGFR).

In vivo, anlotinib has showed broad activity against human tumor xenograft models of the colon (SW-620), ovarian (SK-OV-3), liver (SMMC-7721), renal (Caki-1), glioma (U87MG), and non-small cell lung (Calu-3) during dosing period.

In Sprague-Dawley rats and beagle dogs, anlotinib was rapidly absorbed from the gastrointestinal tracks after oral administration. The oral bioavailability was 23–45 % in rats and 47–74 % in dogs. In vitro metabolism studies demonstrated that anlotinib was primarily metabolized by cytochrome P450-mediated hydroxylation and dealkylation. The oxidized metabolites were excreted directly into the bile or excreted after conjugation mainly to form glucuronides. Anlotinib exhibited large volume of distribution in both species. In rats, primary tissues, such as the lung, kidneys, liver, and heart, exhibited significant higher exposure levels to anlotinib compared with that in plasma. The exposure level in the brain was comparable with the corresponding plasma level. In tumor-bearing mice, anlotinib concentrated 2.4–2.6 times in tumor tissue than in plasma. Besides, anlotinib exhibited in vitro inhibitory activities against human cytochrome P450 3A4 and 2C9 with half maximum inhibitory concentrations of 0.11 and 0.25 μM, respectively.

Based on the promising preclinical antitumor activity, safety, and pharmacokinetics data, this phase I clinical study was performed to determine the dose-limiting toxicity (DLT), maximum tolerated dose (MTD), basic pharmacokinetics, dosage regimen recommended for phase II trial, and preliminary antitumor effects of anlotinib in patients with advanced refractory solid tumor.

## Methods

### Eligibility criteria

Patients with pathologically and/or cytologically proven advanced cancer with no standard therapy were included in this study. Eligibility criteria include ages 18–65, ECOG PS 0–1, and an estimated survival duration of more than 3 months. Patients who had used other chemotherapy drugs need to stop for at least 30 days; patients who had received major surgery needed to rest for at least 4 weeks. Routine blood indices, blood lipids, liver and kidney function, and heart function (left ventricular ejection fraction) are normal; no major organ dysfunction. Patients needed to agree to use and utilize an adequate method of contraception. Patients had to give written informed consent for the study and be willing to comply with criteria of follow-up.

Patients were not eligible if they had the following: brain metastases; spinal cord compression; carcinomatous meningitis; brain or leptomeningeal disease confirmed by CT or MRI examination; urine protein ≥++ confirmed by a 24-h urinary protein excretion >1.0 g; failed to heal wounds or fractures for long term; coagulation abnormalities; experienced arterial or venous thromboembolic events before the first treatment; pre-existing thyroid disease; thyroid function could not be maintained within the normal range under treatment; carried active hepatitis B or hepatitis C; HIV-positive, acquired or congenital immunodeficiency diseases, or organ transplantation; and serious concomitant diseases.

### Study design

This was a first-in-human, phase I, open-label study of anlotinib in advanced refractory solid tumors. The primary objective was to establish the safety profile of anlotinib by identifying DLT, MTD, the recommended phase II dose, and schedule. Secondary objectives included description of single-dose and multi-dose pharmacokinetics of oral anlotinib and assessment of preliminary antitumor effect.

All patients provided written informed consent. The study protocol and amendments were reviewed and approved by the Institutional Review Board, in accordance with the Declaration of Helsinki.

### Treatment

Those patients received escalating doses of anlotinib daily for (1) four consecutive weeks (4/0) or (2) 2-week on/1-week off (2/1). A standard 3 + 3 design was applied, with terms for cohort expansion to six evaluable patients if a DLT (grade 4 blood toxicity or grade 3 neutropenia with fever ≥38.5 °C; grade 3 or higher non-hematologic toxicity) was observed in the first cycle of treatment among the initial three patients. If two DLTs were observed during the first cycle in a cohort, dose-escalation was halted and dose continued at a lower level until the MTD (the highest dose level for which the incidence of first-cycle DLT was <33 %) was identified. In the absence of two or more DLTs in a cohort, the dose was escalated in a modified Fibonacci scheme. Treatment cycles were repeated until disease progression, unacceptable toxicity, or withdrawal of consent.

### Assessment

Efficacies were evaluated according to NCI-proposed Response Evaluation Criteria In Solid Tumors (RECIST 1.1). The tumor will be assessed every two cycles including tumor-related symptoms and physical and imaging examination (CT or MRI) of superficial lesions. Physical and imaging examination of tumor lesions should be done at least four weeks after the first effect evaluation in patients with complete remission (CR), partial remission (PR), and stable disease (SD) to confirm efficacy.

Adverse events are graded into 0–5 according to the National Cancer Institute Common Terminology Criteria for Adverse Events (NCI-CTCAE 4.0). During treatment, blood pressure will be monitored every day. Patients with unresolved adverse reactions at the end of the test needed to be treated and followed until the reactions returned to the grade 1 degree or less, or stable.

### Pharmacokinetic assessments

In single-dose studies, anlotinib were administered orally at dose of 5, 10, 12, or 16 mg anlotinib/person. Serial blood samples were collected in heparinized tubes before and at 0.5, 1, 2, 4, 8, 11, 24, 48, 72, 96, 120, 144, 168, 192, 216, and 240 h (samples of 144–240 h were collected only in 12 or 16 mg/person dose group) after dosing. In the 10 mg/person dose group, urine was sampled before and 0–2, 2–6, 6–11, 11–24, 24–34, 34–48, 48–58, 58–72, 72–96, and 96–120 h after dosing. In multiple-dose studies, there were two ways of multiple-dose administration. Initially, patients in 5 and 10 mg/person/day groups were treated consecutively for 28 days. Blood samples were collected 24 h after dosing on days 1, 2, 4, 7, 10, 13, 15, 16, 18, 21, 24, 28, and 29. Later, the other volunteers were given multiple doses of anlotinib at 10, 12, or 16 mg anlotinib/person/day in the 2/1 schedule for two cycles. Blood samples were collected 24 h after dosing on days 1, 4, 7, 10, 13, 15, 18, 22, 29, and 36. The collected blood samples were centrifuged to prepare plasma fractions; the urine samples were weighed after collection. All samples were stored at −70 °C for subsequent analysis. Concentrations of anlotinib were measured by liquid chromatography/mass spectrometry.

The maximum concentration (*C*_max_) and the time taken to achieve *C*_max_ (*T*_max_) were obtained directly from the data with no interpolation. The area under concentration-time curve up to the last measured time point (AUC_0 − *t*_) was calculated by the trapezoidal rule. The elimination half-life (*t*_1/2_) was calculated using the relationship 0.693/*k*. The *k* was estimated by linear regression analysis of the terminal portion of the log concentration-time data. The renal clearance (CL_R_) was calculated by dividing the cumulative amount excreted into urine (Cum.*A*_*e − U*_) by plasma AUC_0 − *t*_. Accumulation ratio (*R*_ac_) was used to indicate the extent of accumulation during multiple doses of anlotinib capsules and calculated using the following equation:

$$ {R}_{\mathrm{ac}}={C}_{24\kern0.5em \mathrm{h}\left(\mathrm{Day}15\right)}/{C}_{24\kern0.5em \mathrm{h}\left(\mathrm{Day}1\right)}, $$

where *C*_24 h(Day1)_ and *C*_24 h(Day15)_ are plasma concentrations of anlotinib at 24 h after dosing on day 1 and day 15, respectively. Plasma pharmacokinetic (PK) parameters were determined using noncompartmental method with a Kinetica software package (version 5.0; Thermo Scientific, Philadelphia, PA).

### Statistical analysis

The two-side test is used for all statistical tests. *P* ≤ 0.05 is considered as statistical significance. The measurement data will use mean ± SD or median (min, max) for statistical description. Compared with baseline data of screening period, use paired *t* test to compare the difference before/after the treatment within the group. Frequency (constituent ratio) is used for statistical description to the enumeration data. The changes before/after the treatment will use *χ* ^2^ test (accurate probabilistic method) or non-parametric test.

## Results

### Patient characteristics

Thirty-five patients were enrolled in the study between July 2011 and August 2013. Two cohorts of patients received anlotinib at doses ranging from 5 mg (*n* = 4) to 10 mg (*n* = 4) once daily on the 4/0 schedule, and three cohorts of patients at doses of 10 mg (*n* = 3), 12 mg (*n* = 21), and 16 mg (*n* = 3) once daily on the 2/1 schedule. Patient baseline characteristics are presented in Table 1.

**Table 1:** Patient characteristics by anlotinib dose cohort

Characteristic		Number of patients				
		Four consecutive weeks		2-week on/1-week off		
		5 mg/person	10 mg/person	10 mg/person	12 mg/person	16 mg/person
Total		4	4	3	21	3
Sex	Male	2	2	2	15	1
	Female	2	2	1	6	2
Age, year	Median	39	50	60	47	45
	Range	21–49	39–60	53–65	32–60	48–55
ECOG score	0	0	1	0	9	0
	1	4	3	3	12	3
Pretreatment	Surgery	3	4	2	18	2
	Radiotherapy	2	1	3	6	1
	Chemotherapy	4	4	3	15	3
Tumor site	Sarcoma	2	1	1	4	1
	MTC	0	0	0	6	0
	CC	1	2	0	0	1
	NSCLC	0	0	1	3	0
	RC	0	0	0	4	0
	Other	1	1	1	4	1

*ECOG* Eastern Cooperative Oncology Group, *MTC* medullary thyroid carcinoma, *CC* colorectal carcinoma, *NSCLC* non-small cell lung cancer, *RC* renal carcinoma

### Safety and tolerability

On the 4/0 schedule, no DLT was observed in the first four patients at the starting dose of 5 mg/day. However, at 10 mg/day, one patient developed grade 3 hypertension among the first three patients treated. An additional patient was enrolled and also developed grade 3 hypertension. Therefore, the further dose escalation was halted. Meanwhile, PK study revealed a continuously significant anlotinib accumulation in patients who received continuous administration (data not shown). Based on the PK profile of anlotinib and the two DLTs observed at the dose of 10 mg/day, we modified the administration protocol from the 4/0 schedule to the 2/1.

On the 2/1 schedule, because none of the three patients experienced DLT at initial doses of 10 mg/day, the dose escalation proceeded to 16 mg/day. Two of the three patients in the 16 mg cohort experienced DLT (one grade 3 fatigue and one grade 3 hypertension). Therefore, the MTD had been exceeded, and the next lower dose of 12 mg/day was further evaluated by entering additional patients. None of the initial three patients experienced grade 3/4 adverse events. On the basis, 12 mg once daily was selected for the expanding study.

A total of 21 patients received the 12 mg/day dose on the 2/1 schedule. During the first 2 cycles, all the patients experienced an adverse event of any causality. All the hematologic toxicities were mild. As illustrated in Table 2, the most common non-hematologic adverse events were hypothyroidism, triglyceride elevation, total cholesterol elevation, ALT elevation, diarrhea, and proteinuria. During the first 2 cycles, a total of two patients (10 %) experienced grade 3 adverse events (one triglyceride elevation and one lipase elevation). During all treatment cycles, there were six patients (29 %) with grade 3/4 adverse events. The most common (>5 %) non-hematologic grade 3 adverse events were hypertension, triglyceride elevation, hand-foot skin reaction, and lipase elevation.

**Table 2:** Adverse events of patients in 12 mg/day group (the 2/1 schedule)

Adverse events	Grade 1/2				Grade 3			
	First 2 cycles		All cycles		First 2 cycles		All cycles	
	No. of patients	%	No. of patients	%	No. of patients	%	No. of patients	%
Occurred at least one time	21	100	21	100	2	10	6	29
Hand-foot skin reaction	4	19	10	48	0	0	1	5
Rash	4	19	6	29	0	0	0	0
Hypertension	5	24	5	24	0	0	2	10
Proteinuria	5	24	14	67	0	0	0	0
Triglyceride elevation	6	29	11	52	1	5	2	10
Total cholesterol elevation	6	29	13	62	0	0	0	0
Hypothyroidism	8	38	12	57	0	0	0	0
Hyperthyroidism	2	10	2	10	0	0	0	0
ALT elevation	6	29	10	48	0	0	0	0
AST elevation	4	19	9	43	0	0	0	0
Creatinine elevation	1	5	2	10	0	0	0	0
Total bilirubin elevation	5	24	8	38	0	0	0	0
Lipase elevation	1	5	5	24	1	5	1	5
Serum amylase	4	19	9	43	0	0	0	0
Myocardial enzymes abnormal	2	10	3	14	0	0	0	0
Leukopenia	3	14	6	29	0	0	0	0
Neutropenia	0	0	2	10	0	0	0	0
Thrombocytopenia	0	0	2	10	0	0	0	0
Hemorrhage	0	0	1	5	0	0	0	0
Urine occult blood	5	24	8	38	0	0	0	0
Fatigue	5	24	7	33	0	0	0	0
Diarrhea	6	29	7	33	0	0	0	0
Hoarseness	3	14	5	24	0	0	0	0
Nausea	3	14	3	14	0	0	0	0
Inappetence	1	5	2	10	0	0	0	0
Toothache	1	5	4	19	0	0	0	0
Pharyngalgia	1	5	4	19	0	0	0	0
Premature beat	0	0	1	5	0	0	0	0

*ALT* alanine aminotransferase, *AST* aspartate transaminase

### Efficacy

**Fig. 1:**
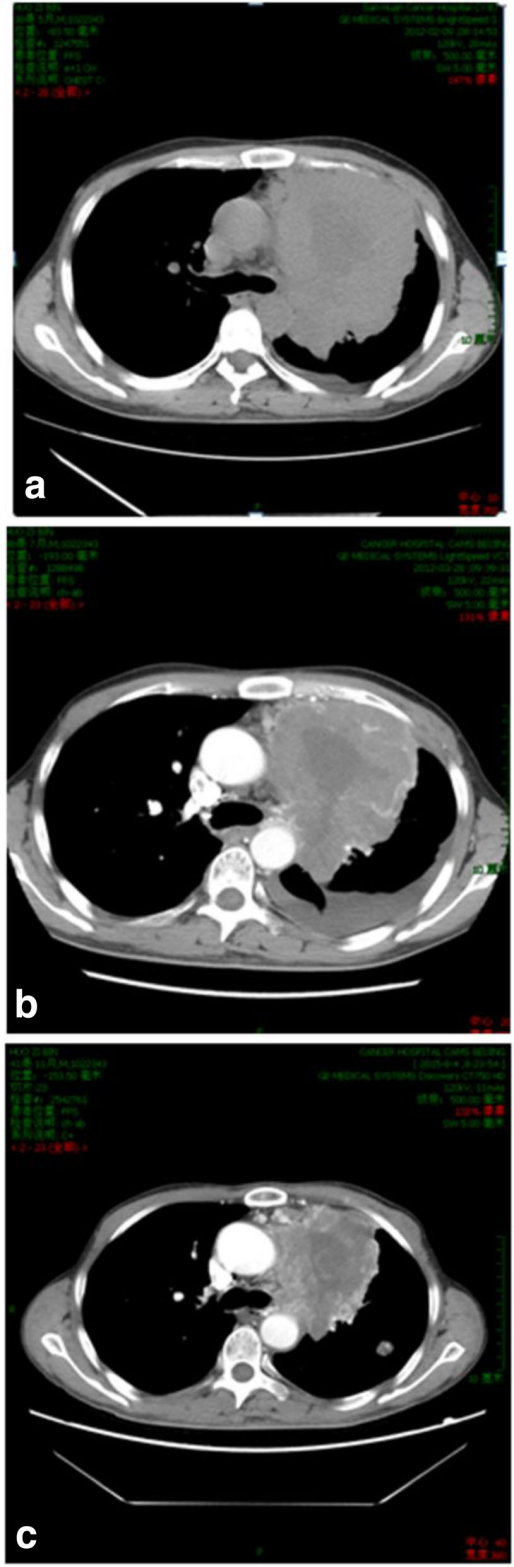
The lung metastasis changes in patients of alveolar soft tissue sarcoma with lung metastasis during treatment. **a** Before treatment. **b** Treatment after 2 cycles (42 days). **c** Treatment after 3 years

According to the RECIST 1.1 criteria, 19 patients had target lesions and one patient with multiple small lung metastases had non-target lesions. Among the 20 patients whose response can be assessed, 3 patients (15 %) had PR; 14 patients (70 %) maintained SD including 12 patients with tumor burden shrinkage, and 3 patients (15 %) had progressive disease (PD) (Additional file 1: Figure S1). PR was observed in the following tumor type: two renal carcinoma and one soft tissue sarcoma. In the current phase I study, other tumor types that responded to anlotinib included medullary thyroid carcinoma, non-small cell lung cancer, colorectal cancer, melanoma, thymic carcinoma, and adenoid cystic carcinoma (Fig. 1).

**Fig. 2:**
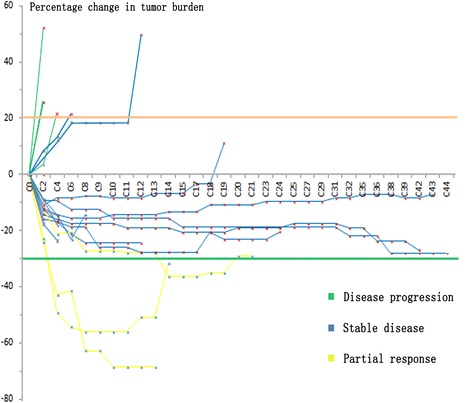
Duration of treatment and tumor size changes of 20 patients who received 12 mg QD at the 2/1 schedule

The median administration duration was 24 weeks. Twenty-four, sixteen, and nine patients had received anlotinib for over 12, 24, and 72 weeks, respectively. Furthermore, the treatment duration had exceeded 100 weeks in three patients (Fig. 2).

### Pharmacokinetics

**Fig. 3:**
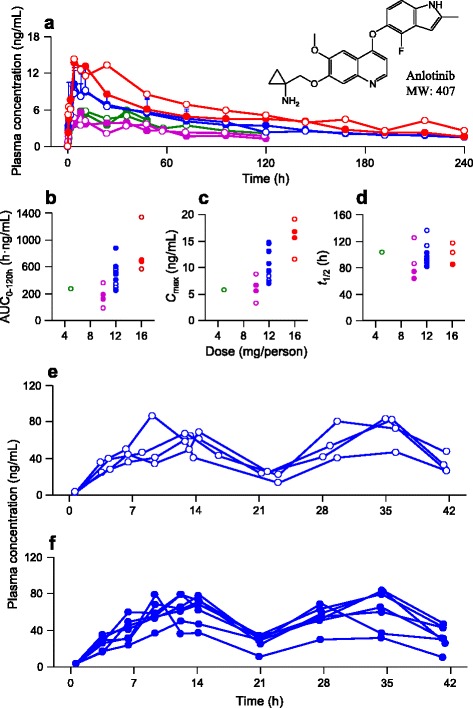
Plasma concentrations of anlotinib over time after a single oral dose of anlotinib capsules at 5 (*green line*), 10 (*purple line*), 12 (*blue line*), or 16 mg anlotinib/person (*red line*) in male (*solid circles*) and female cancer patients (*open circles*) (**a**). **b** Correlation of dose with plasma AUC_0–120 h_. **c** Correlation of dose with plasma *C*_max_. **d** Correlation of dose with *t*_1/2_. **e** Plasma concentrations of anlotinib (24 h after daily dosing) over time during multiple oral doses of anlotinib capsules at 12 mg anlotinib/person/day in female cancer patients. **f** Plasma concentrations of anlotinib (24 h after daily dosing) over time during multiple oral doses of anlotinib capsules at 12 mg anlotinib/person/day in male cancer patients

The mean plasma level-time curve of anlotinib after a single oral administration at 5, 10, 12, or 16 mg/subject is shown in Fig. 3. The associated plasma PK parameters are summarized in Table 3. Anlotinib exhibited rapid intestinal absorption. This was indicated by its plasma concentration, which significantly increased at 1 h after dosing in most patient subjects (Fig. 3). The levels of systemic exposure to anlotinib (*C*_max_ and AUC_0–120h_) tended to increase as the dose level increased from 5 mg anlotinib/person to 16 mg anlotinib/person, but the dose proportionality was inconclusive (Table 3). Anlotinib reached its maximum plasma concentration with *T*_max_ of 4–11 h after dosing, then it eliminated slowly with *t*_1/2_ of 64–136 h and MRT of 124–167 h. The renal excretion of anlotinib was poor, with a CL_R_ of 0.004 L/h/kg; the fraction of the dose excreted into urine (*f*_*e − U*_) was ~0.9 %.

**Table 3:** Pharmacokinetic parameters of anlotinib after a single oral dose of anlotinib capsules in cancer patients and summarized results from dose proportionality assessment

Pharmacokinetic parameters	Measurement of dose-dependent pharmacokinetics				Assessment of dose proportionality			
	5 mg/person (*n* = 1)	10 mg/person (*n* = 4)	12 mg/person (*n* = 11)	16 mg/person (*n* = 4)	*r*	*P*	Slope (90 % CI)	Conclusion
*C*_max_, ng/mL	5.8	5.8 ± 2.8	10.5 ± 2.9	15.8 ± 3.2	0.629	0.003	1.12 (0.54–1.71)	Inconclusive
*T*_max_, h	11.0	6.0 ± 4.4	7.3 ± 3.3	11.0 ± 8.9	–	–	–	–
AUC_0–120h_, h·ng/mL	411	318 ± 133	617 ± 194	894 ± 305	0.465	0.045	0.81 (0.16–1.45)	Inconclusive
AUC_0–∞_, h·ng/mL	687	562 ± 328	1066 ± 263	1585 ± 470	0.666	0.002	1.18 (0.62–1.73)	Inconclusive
*t*_1/2_, h	102	95 ± 22	116 ± 47	98 ± 15	–	–	–	–

Critical intervals were 0.791–1.209 for the systemic exposure data of anlotinib from a single oral dose of anlotinib capsules (10–16 mg) in cancer patients. The term *r* denotes the correlation coefficient. Correlations were statistically significant with *P* < 0.05. The term “linear” was concluded statistically if the 90 % confidence interval (90 % CI) for the slope was contained completely within the critical interval; inconclusive was concluded statistically if the 90 % CI lies partly within the critical interval; nonlinear was concluded statistically if the 90 % CI was entirely outside the critical interval

*C*_max_ maximum plasma concentration, *T*_max_ the time taken to achieve the maximum plasma concentration, *AUC*_0–120h_ the area under concentration-time curve up to 120 h, *AUC*_0–∞_ area under concentration-time curve up to infinity, *t*_1/2_ terminal elimination half-life

Notably, anlotinib exhibited a quite long *t*_1/2_ (96 ± 17 h), which appeared to be dose-independent. The long *t*_1/2_ resulted in significant accumulation of plasma anlotinib over time with a mean *R*_ac_ of 12 ± 7, suggesting drug accumulation over time (Fig. 3). The trough plasma concentration-time curve of anlotinib after treatment of anlotinib at 10, 12, or 16 mg/subject in the 2/1 schedule is depicted in Fig. 3. A 2-week subchronic dosing brought about continuously increased plasma concentration of anlotinib in patients, while its maximum plasma concentration was reached at day 14. Subsequently, the plasma level of anlotinib was apparently decreased with a 7-day washout until the beginning of the other treatment cycle.

## Discussion and conclusions

This study evaluated the multi-targeted tyrosine kinase inhibitor anlotinib in patients with refractory advanced solid tumors and has met its primary objective of establishing the safety profile of anlotinib and identifying a recommended 2/1 schedule and 12 mg-once-daily dose for further investigation in phase II studies.

The toxicity profile of anlotinib was in agreement with that reported in other analogous agents such as sorafenib, sunitinib, and regorafenib [18–20]. The most frequent serious adverse events observed in this study were hand-foot skin reaction, hypertension, fatigue, and lipase elevation. Interestingly, some phase IV studies identified diarrhea as one of the most common serious adverse events in oral anti-VEGFR TKIs [18, 21, 22], but in the present study, 33 % of patients had moderate diarrhea and none developed grade 3 or 4 diarrhea, suggesting the potential of anlotinib in decreasing gastrointestinal toxicity.

In addition, we observed high frequency of triglyceride and cholesterol elevation in this study. Although not resulting in uncomfortable symptoms, they still needed regular monitor because of their close association with arterial thromboembolic event, which was significantly more common in patients receiving anti-VEGFR TKIs [23]. All the adverse events appeared to be manageable even in those patients who received anlotinib for an extended period. Our data supported the close monitor of patients who received anlotinib in terms of lipid and blood pressure status in the following studies.

Although efficacy was not the primary endpoint of phase I study, the tumor response data suggested a substantial and broad antitumor activity of anlotinib. More than 60 % of patients who received 12 mg once daily had tumor burden shrinkage, and the benefit was long-lasting in several patients. Response to anlotinib was noted in a wide range of tumor types, including renal carcinoma, soft tissue sarcoma, medullary thyroid carcinoma, non-small cell lung cancer, colorectal cancer, melanoma, thymic carcinoma, and adenoid cystic carcinoma. This remarkable antitumor activity strongly justified the conduct of phase II clinical trials in those tumor types.

PK assessments in this study demonstrated that anlotinib is rapidly absorbed through the intestine and eliminated slowly with a half-life of 96 h, which resulted in significantly continuous accumulation of anlotinib in plasma over time. This is in agreement with the high frequency of grade 3 toxicity at 10 mg in the 4/0 schedule. To adapt to this PK feature, we changed the administration protocol to the 2/1 schedule, where we observed a 2-week subchronic rise in plasma concentration with the peak at day 14, and an apparent decline during the 7-day washout until the beginning of next cycle. This indicated the rational extension of administration interval is more conductive to the establishment of optimal dose for drug with long elimination half-life period.

Based on the toxicity and efficacy profile, anlotinib displayed manageable toxicity and broad-spectrum antitumor potential. The recommended treatment regimen for the following clinical studies is as follows: anlotinib monotherapy, 12 mg per day, on the 2/1 schedule. Based on the promising results in phase I study, a series of phase II clinical trials have been performed and initial efficacy has been observed in several types of solid tumor including colon adenocarcinoma, non-small cell lung cancer, renal clear cell cancer, medullary thyroid carcinoma, and soft tissue sarcoma (details pending publication elsewhere).

## Additional file

Additional file 1: Figure S1. Waterfall plot of best-percent change in target lesions from baseline for the 19 patients who had target lesions on the basis of investigator assessment. (PDF 287 kb)

## Abbreviations

ALT: Alanine aminotransferase
AUC: Area under concentration-time curve
CR: Complete remission
DDR1: Discoidin domain receptor 1
DLT: Dose-limiting toxicity
EGFR: Epidermal growth factor receptor
FGF: Fibroblast growth factor
FGFR: Fibroblast growth factor receptor
MTD: Maximum tolerated dose
NCI-CTCAE: National Cancer Institute Common Terminology Criteria for Adverse Events
PD: Progressive disease
PDGFR: Platelet-derived growth factor receptor
PK: Pharmacokinetic
PR: Partial remission
RECIST 1.1: Response Evaluation Criteria In Solid Tumors (version 1.1)
SD: Stable disease
TKI: Tyrosine kinase inhibitor
VEGF: Vascular endothelial growth factor
VEGFR: Vascular endothelial growth factor receptor
