# Supplementary material for: Safety, pharmacokinetics, and antitumor properties of anlotinib, an oral multi-target tyrosine kinase inhibitor, in patients with advanced refractory solid tumors
Source: J Hematol Oncol. 2016 Oct 4;9:105. doi: 10.1186/s13045-016-0332-8 (PMC5051080; doi:10.1186/s13045-016-0332-8)
Supplement: Additional file 1: Figure S1. — Waterfall plot of best-percent change in target lesions from baseline for the 19 patients who had target lesions on the basis of investigator assessment. (PDF 287 kb) [file 13045_2016_332_MOESM1_ESM.pdf]

Safety, pharmacokinetics, and antitumor properties of anlotinib, an oral multi-target tyrosine kinase inhibitor, in patients with advanced refractory solid tumors

Yongkun Sun, Wei Niu, Feng Du, Chunxia Du, Shuting Li, Jinwan Wang, Li Li, Fengqing Wang, Yu Hao, Chuan Li and Yihebali Chi

Journal of Hematology & Oncology (DOI 10.1186/s13045-016-0332-8)

Additional file 1

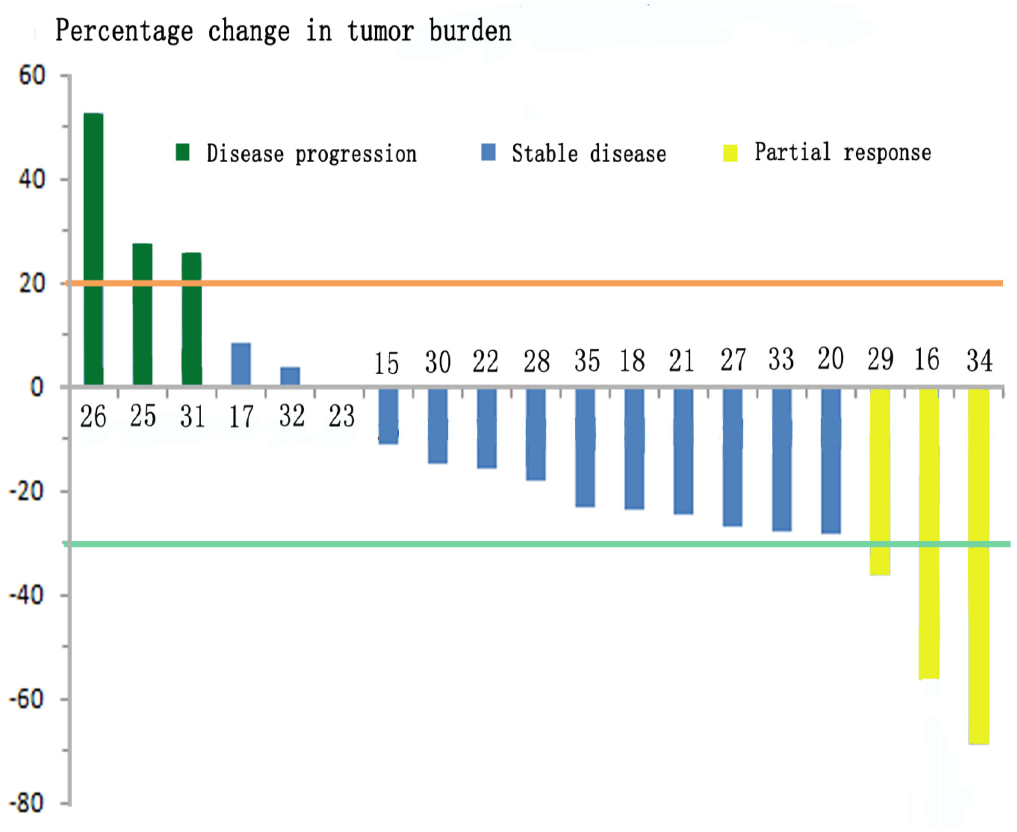

Fig. S1. Waterfall plot of best-percent change in target lesions from baseline for the 19 patients who had target lesions on the basis of investigator assessment
